# The SR protein RSP-2 influences expression of the truncated insulin receptor DAF-2B in *Caenorhabditis elegans*

**DOI:** 10.1093/g3journal/jkad064

**Published:** 2023-03-26

**Authors:** Bryan A Martinez, Matthew S Gill

**Affiliations:** Institute on the Biology of Aging and Metabolism and the Department of Genetics, Cell Biology and Development, University of Minnesota, 4-114 Nils Hasselmo Hall, 312 Church Street SE, Minneapolis, MN 55455, USA; Institute on the Biology of Aging and Metabolism and the Department of Genetics, Cell Biology and Development, University of Minnesota, 4-114 Nils Hasselmo Hall, 312 Church Street SE, Minneapolis, MN 55455, USA

**Keywords:** splicing, dauer larva, insulin, aging, pheromone

## Abstract

The alternatively spliced *daf-2b* transcript in *Caenorhabditis elegans* encodes a truncated isoform of the nematode insulin receptor that retains the extracellular ligand binding domain but lacks the intracellular signaling domain and is therefore unable to transduce a signal. To identify factors that influence expression of *daf-2b*, we performed a targeted RNA interference screen of *rsp* genes, which encode splicing factors from the serine/arginine protein family. Loss of *rsp-2* significantly increased the expression of a fluorescent *daf-2b* splicing reporter, as well as increasing expression of endogenous *daf-2b* transcripts. Correspondingly, *rsp-2* mutants exhibited similar phenotypes to those previously observed with DAF-2B overexpression, namely suppression of pheromone-induced dauer formation, enhancement of dauer entry in insulin signaling mutants, inhibition of dauer recovery, and increased lifespan. However, the epistatic relationship between *rsp-2* and *daf-2b* varied according to the experimental context. Increased dauer entry and delayed dauer exit of *rsp-2* mutants in an insulin signaling mutant background were partially dependent on *daf-*2b. Conversely, suppression of pheromone-induced dauer formation and increased lifespan in *rsp-2* mutants were independent of *daf-2b.* These data demonstrate that *C. elegans* RSP-2, an ortholog of human splicing factor protein SRSF5/SRp40, is involved in regulating the expression of the truncated DAF-2B isoform. However, we also find that RSP-2 can influence dauer formation and lifespan independently of DAF-2B.

## Introduction

Alternative splicing is a highly regulated process that increases protein diversity (Nilsen and Graveley 2010). Multiple mechanisms of splicing lead to variations in mRNA transcripts from pre-mRNA pools. These changes are governed by combinatorial interactions of regulatory proteins that form the spliceosome and include alternate exon inclusion or exclusion, intron retention, use of alternative 5′ and 3′ splice sites, differences in 5′ and 3′ UTR length, as well as differential use of polyadenylation sites (Zahler 2012). In mammals, it is well established that alternative splicing of the insulin receptor (IR) yields 2 isoforms that arise from the exclusion (IR-A) or inclusion (IR-B) of a short cassette exon (Belfiore *et al*. 2009). The *C. elegans* IR, encoded by the *daf-2* gene, also undergoes a similar alternative splicing process to yield homologous full-length isoforms DAF-2A/IR-A and DAF-2C/IR-B (Ohno *et al*. 2014). In addition, we have characterized another alternatively spliced DAF-2 isoform (DAF-2B) that is predicted to arise via activation of an intronic polyadenylation site (Martinez *et al*. 2020). This isoform retains the extracellular ligand-binding domain but lacks the intracellular signaling domain and functions as a decoy receptor to regulate insulin signaling in a manner that is dependent on the prevailing insulin peptide milieu (Martinez *et al*. 2020).

The *daf-2a* transcript is encoded by 17 exons and *daf-2c* arises by the inclusion of exon 11.5 between exons 11 and 12 (Ohno *et al*. 2014). The activation of an intronic polyA site downstream of exon 11.5 is predicted to lead to expression of the *daf-2b* transcript through inhibition of the exon 11.5 5′ splice site, transcript truncation through alternative 3′ end formation, and finally stabilization through polyadenylation. This process of intronic polyA activation has been described for mammalian receptor tyrosine kinases including the IR (Vorlová *et al*. 2011) suggesting that is a conserved regulatory mechanism. Although the functional consequences of the truncated mammalian IR have not been described, a truncated transcript of the PDGF receptor has been shown to act as a soluble decoy receptor (Mueller *et al*. 2016).

During development, the insulin signaling pathway in *C. elegans* is involved in regulating the decision to proceed with reproductive growth or enter a diapause stage called the dauer larva. Reduced insulin signaling arising from hypomorphic *daf-2* mutations or loss of agonist insulin peptides leads to temperature-dependent dauer arrest and increased lifespan (Kenyon *et al*. 1993; Fernandes de Abreu *et al*. 2014). We previously showed that overexpression of DAF-2B enhances dauer formation in mutants with reduced insulin signaling by sequestering agonist insulin peptides and further decreasing insulin signaling (Martinez *et al*. 2020). Moreover, overexpression of DAF-2B in adult animals leads to increased lifespan. In the natural environment, a secreted pheromone promotes dauer formation in conjunction with reduced food availability and elevated temperatures. In this context, overexpression of DAF-2B suppresses dauer formation via sequestration of antagonist insulin peptides (Martinez *et al*. 2020).

Splicing factors from the serine arginine (SR) protein family are involved in multiple aspects of alternative splicing such as spliceosome assembly, constitutive and alternative splicing of pre-mRNA, exon identity, mRNA export as well as polyadenylation site selection (Zahler 2012). SR proteins are characterized by an RNA recognition motif (RRM) that facilitates mRNA binding and a serine/arginine-rich C-terminus that is involved in protein-protein interactions (Jeong 2017). There are 8 members of the SR protein family in *C. elegans*, encoded by *rsp* genes, but relatively little is known about their function or splicing targets. RNA interference (RNAi) studies of *rsp* genes revealed that only *rsp-3* is an essential gene (Kawano *et al*. 2000; Longman *et al*. 2000, 2001), and a role for other *rsp* genes in alternative splicing has been confirmed (Barberan-Soler *et al*. 2011). Interestingly, *rsp-2* was identified as a strongly regulated dauer gene (Liu *et al*. 2004), suggesting that changes in its own expression and splicing of its targets could contribute to dauer formation.

We have previously used an in vivo fluorescent splicing reporter strain to demonstrate temporal and spatial regulation of *daf-2b* splicing capacity, suggesting that *daf-2b* is an inducible transcript whose expression is likely to be driven by the activity of developmentally regulated splicing factors. In this study, we set out to determine if SR proteins play a role in regulating the expression of DAF-2B by performing a targeted RNAi screen of the *C. elegans rsp* genes and found that loss of *rsp-2* led to increased expression of the DAF-2B splicing reporter. Genetic analysis using viable *rsp-2* mutants indicated that loss of *rsp-2* also increased endogenous *daf-2b* transcripts. Although *rsp-2* knockouts exhibited the same phenotypes as DAF-2B overexpression, the epistatic relationship between *rsp-2* and *daf-2b* differed for each phenotype. For instance, suppression of pheromone-induced dauer formation and increased longevity in *rsp-2* mutants were independent of DAF-2B, whereas the effects of *rsp-2* deletion on dauer formation and dauer recovery in mutants with reduced insulin signaling partially required DAF-2B. These findings indicate that one function of the RSP-2 splicing factor is to inhibit DAF-2B expression but RSP-2 also influences pheromone-induced dauer formation and lifespan independently of DAF-2B.

## Materials and methods

### Plasmids

All primers and plasmids are listed in Supplementary Tables 1 and 2 in Supplementary File 1. To generate an *rsp-2* genomic rescue plasmid, 1 kb of the *rsp-2* promoter, amplified with *Sph*I and *Age*I overhangs (primers 1 & 2), and the genomic *rsp-2* coding sequence, amplified with *Age*I and *Kpn*I overhangs (primers 3 and 4), were cloned into pMGL99 cut with *Sph*I and *Kpn*I to generate pMGL221[*rsp-2p::RSP-2*]. The resulting plasmid was sequenced (primer 5) to confirm the correct *rsp-2* sequence.

### *Caenorhabditis elegans* strains and maintenance

*C. elegans* strains were maintained as previously described (Brenner 1974). Bristol N2 (wild-type), VC463[*rsp-2(ok639) II*], and JT709[*pdk-1(sa709) X*] were obtained from the *C. elegans* Genetics Center (University of Minnesota, MN). MGL264[*jluIs15[daf-2p::DAF-2bexon-11.5::tdTomato* + *rol-6(+)*] (Martinez *et al*. 2020) was outcrossed twice to generate MGL371. All worm strains used in the study are listed in Supplementary Table 3 in Supplementary File 1.

### Genetic crosses

Genetic crosses were performed using standard methods. When using mutant strains generated from mutagenesis, wild type control strains were derived from the final outcross with N2. After outcrossing, strains generated by CRISPR were compared to the lab wildtype N2 background.

VC463 was genotyped with primers 6–8 and was outcrossed 5 times with N2 to generate MGL372[*rsp-2(ok639)*] and a wild-type control, MGL460. For clarity, the deletion mutant *rsp-2(ok639)* is referred as *rsp-2(Δ).* MGL372 was crossed into the *daf-2b::tdTomato* reporter strain MGL371 to generate MGL373[*rsp-2(ok639); jluIs15[daf-2p::DAF-2bexon-11.5::tdTomato* + *rol-6(+)*]. The temperature sensitive dauer constitutive (Daf-c) phenotype at 27°C was used to confirm the presence of *pdk-1(sa709).* Since *pdk-1* is on the X chromosome, we crossed hemizygous F1 males from a cross back into the *pdk-1* parental line to ensure *pdk-1* homozygosity before segregating heterozygotes for the gene of interest. In this way, JT709 was outcrossed 3 times to generate MGL374, which was then crossed into MGL372 to generate MGL375[*rsp-2(ok639); pdk-1(sa709)*] and the corresponding *pdk-1* control MGL461.

We previously generated a *daf-2b* deletion strain that carries a *daf-2bc* deletion and is rescued for *daf-2c* by a single copy insertion of *daf-2c* cDNA at the ttTi5605 Mos insertion locus (Martinez *et al*. 2020). The *daf-2c* Mos insertion is located on chromosome II within 2.7 map units of the *rsp-2* locus. We therefore crossed MGL372[*rsp-2(ok639)*] with the *daf-2c* MosSCI strain MGL297[*jluSi3[daf-2p::DAF-2C* + *unc-119(+)]*] and identified heterozygous recombinants in order to generate MGL376[*rsp-2(ok639) jluSi3[daf-2p::DAF-2C* + *unc-119(+)]*]. This strain was then crossed with MGL302[*jluSi3[daf-2p::DAF-2C* + *unc-119(+)]; daf-2(jlu1)*] to generate MGL377[*rsp-2(ok639) jluSi3[daf-2p::DAF-2C* + *unc-119(+)]; daf-2(jlu1)*]. *daf-2b* deletion strains were genotyped using primers 9–15. To examine *rsp-2* and *daf-2b* epistasis in the *pdk-1* background, we first crossed MGL374 into MGL297 and MGL377 to generate MGL378[*jluSi3[daf-2p::DAF-2C* + *unc-119(+)]; pdk-1(sa709)*] and MGL379[*rsp-2(ok639) jluSi3[daf-2p::DAF-2C* + *unc-119(+)]; daf-2(jlu1); pdk1(sa709)*], respectively. MGL378 and MGL379 were crossed together to derive MGL380[*rsp-2(ok639) jluSi3[daf-2p::DAF-2C* + *unc-119(+)]; pdk-1(sa709)*] and MGL381[*jluSi3[daf-2p::DAF-2C* + *unc-119(+)]; daf-2(jlu1); pdk-1(sa709)*].

### Transgenic strains

Strains expressing wild-type *rsp-2* genomic rescue constructs were generated by microinjection of pMGL221 into MGL371[*rsp-2(ok639)*] and MGL373[*rsp-2(ok639); pdk-1(sa709)]*. Each construct was injected at a concentration of 10 ng/μL with a *myo-3p::GFP* coinjection marker at a concentration of 25 ng/μL. Three independent lines were analyzed for each construct in each background.

To examine the effects of DAF-2B overexpression in the *pdk-1* mutant background, we first generated a transgenic line, MGL462*[jluEx186(daf-2p::DAF-2B* + *myo-2p::tdTomato)]*, expressing extrachromosomal DAF-2B under the native *daf-2* promoter by injecting *daf-2b* cDNA at 25 ng/µL with 5 ng/uL *myo-2p::tdTomato* coinjection marker. This line was integrated with UV and outcrossed 6 times to N2 to generate MGL450[*jluIs18*(*daf-2p::DAF-2B* + *myo-2p::tdTomato*)]. MGL450 was crossed with MGL374 to generate MGL451*[jluIs18(daf-2p::DAF-2B* + *myo-2p::tdTomato) pdk-1(sa709)]*.

### CRISPR gene editing

For all CRISPR edits, we used the co-CRISPR approach of Paix *et al.* (Paix *et al*. 2015), in which the *dpy-10* locus is edited using a *dpy-10* Cas-9 CRISPR RNA (crRNA) (oligo 16) and a *dpy-10* homology-directed repair (HDR) template (oligo 17), synthesized as a single-stranded oligo. Recombinant Cas9 was purified from *Escherichia coli* according to the method of Paix *et al.* (Paix *et al*. 2015). Single stranded repair templates were synthesized by Eurofins. crRNA and trans-activating crRNA (tracrRNA) were obtained from Dharmacon and Integrated DNA Technologies (IDT).

Injection mixes were prepared according to Paix *et al.* (Paix *et al*. 2015) (transcriptional reporter) or Wang *et al.* (Wang *et al*. 2018) (null mutant). For all edits, ∼30 animals were injected and singled onto individual plates. On the first day of adulthood, F1 jackpot plates were identified by the presence of large numbers of *dpy-10* rollers, and 16 animals were singled from each of 2 or 3 jackpots to new plates. F1 animals were genotyped for the presence of the edit, and progeny were cloned out to identify F2 homozygotes. All edits were confirmed by direct sequencing before proceeding with subsequent experiments.

### *rsp-2* CRISPR null mutant

CRISPR with HDR was used to generate a null mutant of *rsp-2* using the method of Wang *et al.* (Wang *et al*. 2018). A crRNA targeting a protospacer adjacent motif (PAM) sequence close to the ATG start site of the *rsp-2* coding sequence (oligo 18) was identified using Wormbase (Au *et al*. 2019). A single-stranded DNA repair template consisting of 35 bp of *rsp-2* sequence flanking a 43 bp universal stop knock-in (STOPIN) cassette was synthesized (oligo 19). F1 animals were genotyped for the presence of the STOPIN cassette using primers 20 and 21, and progeny were cloned out to identify STOPIN homozygotes (primers 22 & 23). Two independent STOPIN lines were identified and sequenced for correct insertion of the knock-in cassette (primers 20, 8, 22, and 23). One line was outcrossed twice to N2, and the resulting strain MGL400[*rsp-2(jlu14)*] was used for subsequent analyses. MGL400 was crossed into the *pdk-1* mutant to generate MGL401[*rsp-2(jlu14); pdk-1(sa709)*]. For clarity, *rsp-2(jlu14)* is referred to as *rsp-2(STOPIN)*.

### *rsp-2* CRISPR transcriptional reporter

We used a modification of the SKI LODGE approach (Silva-García *et al*. 2019) to generate a single copy *rsp-2* transcriptional reporter inserted at a safe harbor site. crRNA targeting the PAM site associated with the ttTi4348 MosSCI locus on chromosome I was synthesized (oligo 24 [Silva-García *et al*. 2019]). We used in vivo assembly of the HDR template (Paix *et al*. 2016) to insert the *rsp-2* reporter construct into the ttTi4348 locus. 1 kb of *prsp-2* sequence was amplified from genomic DNA with a 35 bp 5′ ttTi4348 homology region and a 35 bp 3′ mNeonGreen homology region (primers 25 and 26). mNeonGreen was amplified from pDG353 (Hostettler *et al*. 2017) with a 35 bp 5′ *prsp-2* homology region and a 35 bp 3′ *rsp-2* 3′UTR homology region (primers 27 and 28). The *rsp-2* 3′ UTR was amplified from genomic DNA with primers that added a 35 bp 5′ mNeonGreen homology region and a 35 bp 3′ ttTi4348 homology region (primers 29 and 30). F1 animals were genotyped for the presence of the reporter construct using primers 31–33, and progeny were cloned out for identification of homozygotes. Two independent lines were identified and sequenced for the correct insertion of the reporter construct (primers 31, 32 and 34). One line was outcrossed twice to N2, and the resulting strain MGL406[*jluSi5*(*rsp-2p::mNeonGreen::rsp-2 3′UTR) I*] was used for subsequent analyses. MGL406 was crossed into the *pdk-1* mutant to generate MGL407[*jluSi5*(*rsp-2p::mNeonGreen::rsp-2 3′UTR) I; pdk-1(sa709) X*].

### RNAi screen of *rsp* genes

RNAi clones were obtained from the Open Biosystems RNAi Library and sequenced to confirm their identity. RNAi bacteria were grown in the presence of tetracycline (5 µg/mL) and ampicillin (100 µg/mL) antibiotics, and single colonies were inoculated in LB with ampicillin (100 µg/mL) for 16–20 hours at 37°C. Nematode growth medium (NGM) plates containing 1 mM IPTG and 100 µg/mL ampicillin were seeded with an overnight culture of bacteria, dried in a sterile hood, and maintained at room temperature overnight.

Eggs from hypochlorite treatment of gravid *daf-2b::tdTomato* adults were seeded onto RNAi plates and maintained until animals reached adulthood. Ten to fifteen gravid adults were then transferred to fresh RNAi plates, allowed to lay eggs for 2 hours, and removed. We then scored fluorescence qualitatively at each developmental stage starting at the L2 stage on triplicate plates for each clone. Each plate was given a qualitative score of 0 (no change), 1 (a minor increase in fluorescence), 2 (noticeably increased fluorescence), or 3 (robust and intense increase in fluorescence). As previously described, RNAi of *rsp-3* and *rsp-7* caused developmental defects in the second generation of exposure (Kawano *et al*. 2000; Longman *et al*. 2000; Kamath *et al*. 2003; Simmer *et al*. 2003; Sönnichsen *et al*. 2005). For these clones, animals were grown on L4440 control bacteria for the first generation and then used for an egg lay on the *rsp* RNAi bacteria.

### Fluorescence imaging and quantitation

**Fig. 1.**
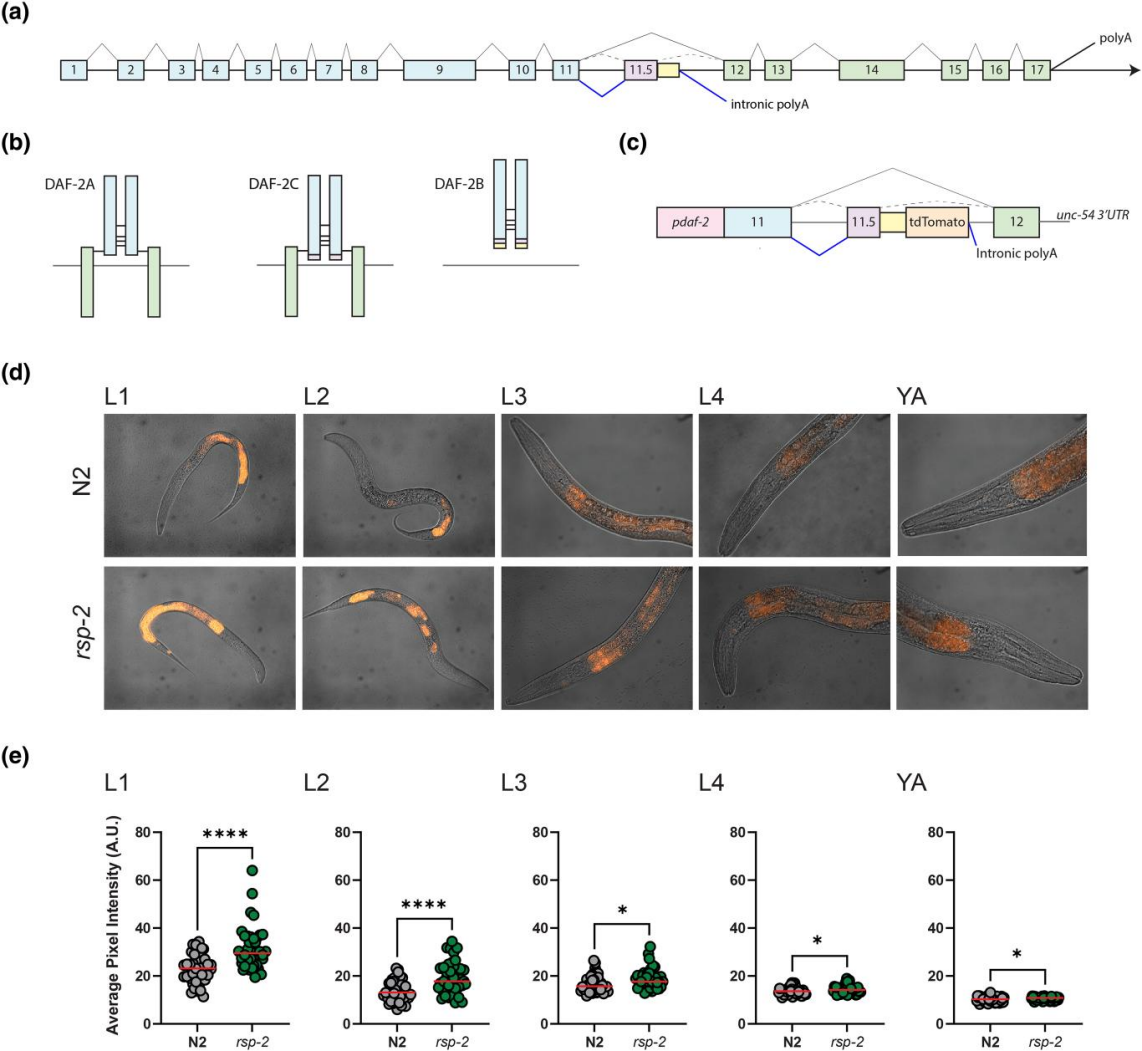
Genetic deletion of *rsp-2* increases expression of a *daf-2b* splicing reporter. a) Organization of the *daf-2* genomic locus. Exons 1–11 encode the extracellular domain (α subunit) and 12–17 encode the transmembrane an intracellular domain (β subunit). Splicing of exons 1 to 17 generates the *daf-2a* transcript, while inclusion of exon 11.5 yields the *daf-2c* transcript. Intronic polyA activation produces the *daf-2b* transcript which lacks exons 12–17. b) Predicted protein products of DAF-2A, DAF-2C and DAF-2B. c) Structure of the DAF-2B splicing reporter. Activation of the intronic polyA site downstream of exon 11.5 5′splice site yields a fluorescent product. d) Representative fluorescence images showing the expression of the DAF-2B::tdTomato splicing reporter through reproductive development in wild type (N2) worms and increased expression in the *rsp-2(Δ)* background. e) Quantitation of DAF-2B::tdTomato expression through development. Data are derived from 20 images per genotype at each developmental stage. Student's *t*-test ****P < 0.0001, ***P < 0.001, **P < 0.01.

For quantitation of DAF-2B::tdTomato expression, images were obtained at each developmental stage using a Zeiss AxioCam Icm 1 monochromatic camera and AxioVision imaging software on a Zeiss Axio Observer A1 inverted microscope with objective lenses ranging from 10 × to 40x. For each developmental stage, 20–30 images were taken with identical exposure times and camera settings (L1 and L2: 40×, 200 ms; L3 and L4: 20×, 500 ms; YA: 10×, 500 ms). TIFF images were analyzed using the free-selection tool on ImageJ to trace the perimeter around the fluorescent area of the worm. This fluorescent area is generally from the tail to the anterior region of the intestine. The average pixel intensity of the area was compared between groups. For Fig. 1, representative images were generated using the manual-multicolor pseudocolor merge function through the Zeiss AxioVision software.

Analysis of the *rsp-2* transcriptional reporter was performed in the same way but the entire animal was selected as the region of interest. Late L1 animals were analyzed at 16 hours after egg lay under conditions defined for both pheromone and reduced insulin signaling dauer formation. For experiments involving comparison of L3, L2D, and dauer animal populations, plates were maintained for 30–36 hours. At these time points L3 animals can be discerned morphologically from L2D animals based on size, fat deposition, and animal shape. Once sufficient L3 and L2D animals were taken from the population for imaging, these plates were returned to their respective temperature until 50–60 hours posthatching, after which dauer animals were isolated and measured.

### RNA extraction and generation of cDNA

Synchronous populations of wildtype or *rsp-2(ok639)* L1 animals were generated for RNA extraction by hypochlorite treatment. Approximately 60,000 animals were harvested from 6 independent populations per strain. After washing in S-basal, populations were subjected to freeze-thaw prior to sonication (10 s max, 3 times) using a 130 Watt 20 kHz Ultrasonic Processor and 2 mm stepped microtip. Samples were retained on ice for a maximum of 30 minutes. Total RNA was extracted using RNAzol RT (Sigma-Aldrich) following manufacturers guidelines. Total RNA pellets were resuspended in water and reprecipitated with lithium chloride (Invitrogen) to remove contaminants and residual DNA. Samples were resuspended in 10 mM Tris, and concentration was measured using a Nanodrop spectrophotometer. 1 ug of RNA was converted to cDNA using the Maxima H-minus First Strand cDNA Synthesis kit (ThermoFisher Scientific) which includes a dsDNAase degradation step. Samples were diluted in 10 mM Tris to 10 ng/uL.

### Quantitative PCR

cDNA samples were analyzed by quantitative PCR using the StepOnePlus Real-time PCR System with SYBR Green qPCR Master Mix (Applied Biosystems). All annealing was performed at 60°C. All conditions were performed with 3 technical replicates. Cycle quantification (Cq) values were measured with StepOnePlus software.

Eight reference genes (Supplementary Table 2 in Supplementary File 1, primers 35–50) were measured in each of our 12 samples (6 samples from each genotype) to determine which were most stable. M-analysis using qBase + (Biogazelle) indicated that the most stable reference genes were *cdc-42* and *pmp-3 (*data not shown), and 2 reference genes were sufficient. Oligos for *daf-2a* (primers 51–52), *daf-2b* (primers 53–54), and *daf-2c* (primers 55–56) were designed to span exon-exon boundaries and were evaluated for efficiency using standard curves across a large concentration range. Relative quantification (RQ) values were derived using the average cycle threshold (Ct) value of the control strain (N2) replicates. Relative gene expression was calculated using Pffafl's formula (Pfaffl 2001) and included the geometric mean of the RQ values derived from the reference genes *cdc-42* and *pmp-3*.

### Dauer assays

Pheromone extraction, dauer entry, and recovery assays were performed as previously described with minor modifications (Martinez *et al*. 2020). For pheromone-induced dauer formation, peptone-free NGM plates with added pheromone extract were seeded with 100 µL of an overnight culture of OP50 *E. coli* resuspended in S-basal and 1 mg/mL ampicillin at a concentration of 3 × 10^9^ cfu/mL S-basal. Eggs from a 2 h lay were maintained at 25°C for 50–60 hours. Dauers were scored on the basis of morphology and expressed as a percentage of the population. For dauer entry assays in the *pdk-1* mutant background, peptone-free NGM plates media were seeded with 150 µL of an overnight culture of OP50 *E. coli* resuspended in S-basal and 1 mg/mL ampicillin at a concentration of 5 × 10^9^ cfu/mL. Eggs from a 2 h lay were maintained at 26.2°C for 48–50 hours. Dauers were scored on the basis of morphology and expressed as a percentage of the population. For transgenic lines, usually 3 but at least 2 independent isolates were utilized and compiled together.

To measure dauer recovery in the *pdk-1* background, dauer larva were generated by incubation at 27°C in the manner described above for dauer entry conditions. Dauers were SDS-selected, and 50–100 animals were placed in the center of a fresh 1.5% agarose (dissolved in S-basal) plate seeded with OP50 in triplicate. Recovery plates were maintained at 25°C and scored at the indicated time points for the presence of nondauers. Animals that never recovered from dauer arrest during the observation window were right-censored. For transgenic lines, usually 3 but at least 2 independent isolates were utilized and compiled together.

### Lifespan assays

Lifespan assays were performed at 20°C on OP50 *E. coli* bacteria on NGM agar plates with a fresh lawn of bacteria with or without 10μg/mL 5-fluoro-2′-deoxyuridine (FUDR). For assays in the absence of FUDR, L4 larvae from a synchronized lay were transferred to a fresh plate and transferred daily during the reproductive period to prevent progeny contamination. Death was scored by loss of touch-provoked movement, and animals lost due to bagging, uterine prolapse, or crawling up the side of the petri dish were censored. Entire experiments were replicated at least twice. Lifespan data was graphed using a Kaplan–Meier format and analyzed using the log-rank test.

### Statistical analysis

The sample size for each experiment was determined empirically and was based on accepted practice within the *C. elegans* field. Statistical analysis was performed using GraphPad Prism v8.0 with *P* < 0.05 indicating significance. For pairwise comparisons the Student's *t-*test without correction was used. For comparisons *k* > 2, 1-way ANOVA followed by a Tukey's post hoc test was used. The log-rank test was used to analyze dauer recovery and lifespan data.

## Results

### Loss of *rsp-2* increases *daf-2b* expression

The *daf-2a* transcript is encoded by 17 exons and *daf-2c* arises by the inclusion of exon 11.5 (Ohno *et al*. 2014) (Fig. 1a). *daf-2b* likely arises via activation of an intronic polyA site downstream of exon 11.5 and the resulting transcript contains a 46 bp extension of exon 11.5 that includes an in-frame stop codon (Fig. 1a). DAF-2A and DAF-2C contain both a ligand binding domain and a transmembrane/signaling domain and the presence of exon 11.5 in the *daf-2c* transcript is predicted to result in a short peptide extension on the ligand binding domain (Fig. 1b). DAF-2B has a further extension on the ligand binding domain but completely lacks the transmembrane and signaling domain (Fig. 1b).

To determine the timing and location of DAF-2B splicing capacity, we previously generated a splicing reporter, which consists of the native *daf-2* promoter upstream of a genomic fragment spanning exons 11 and 12 along with the intervening intronic sequence. To visualize splicing associated with *daf-2b* expression, we inserted the tdTomato fluorescent protein before the in-frame stop codon and upstream of the intronic polyA site (Martinez *et al*. 2020) (Fig. 1c). To identify splicing factors that increase the expression of the *daf-2b* transcript, we used RNAi to knock down the *C. elegans* orthologs of the SR protein family (*rsp-1* through *rsp-8*) in this *daf-2b*-specific splicing reporter strain. Initially, DAF-2B::tdTomato fluorescence was scored qualitatively, relative to the L4440 empty vector control (0—no increase, 1—weak increase, 2—increase, and 3—strong increase), and an expression score was assigned from triplicate plates per RNAi clone. Knockdown of *rsp-2* by RNAi resulted in a consistent increase in DAF-2B::tdTomato fluorescence in early larval stages that declined into adulthood, while other *rsp* family members showed little to no increase (Table 1). Subsequently, the effect of *rsp-2* RNAi was confirmed with a more detailed analysis using quantitative fluorescence imaging (Supplementary Fig. 1 in Supplementary File 1). To further confirm that loss of *rsp-2* influences DAF-2B::tdTomato expression, we obtained an *rsp-2* deletion (Δ) mutant and crossed it into the DAF-2B::tdTomato reporter background. We observed a similar increase in DAF-2B::tdTomato fluorescence in the *rsp-2(Δ)* background using quantitative fluorescent measurements (Fig. 1d and e).

**Table 1.** Effect of *rsp* RNAi on expression of a *daf-2b* splicing reporter.

Gene	L2 Score	L3 score	L4 score	YA score	Total score
*rsp-1*	2	1	0	0	3
*rsp-2*	5	4	4	1	14
*rsp-3^a^*	0	0	0	0	0
*rsp-4*	0	1	0	0	1
*rsp-5*	0	0	0	2	2
*rsp-6*	0	0	0	0	0
*rsp-7^a^*	1	1	2	0	4
*rsp-8*	1	0	2	0	3

RNAi was performed in triplicate for each larval stage. Individual plates were scored qualitatively and assigned a score relative to controls grown on L4440 (0—no increase, 1—weak increase, 2—increase and 3—strong increase). The sum of the scores across triplicate plates is presented in the table.

Scored in first generation due to lethality/arrest in second generation.

**Fig. 2.**
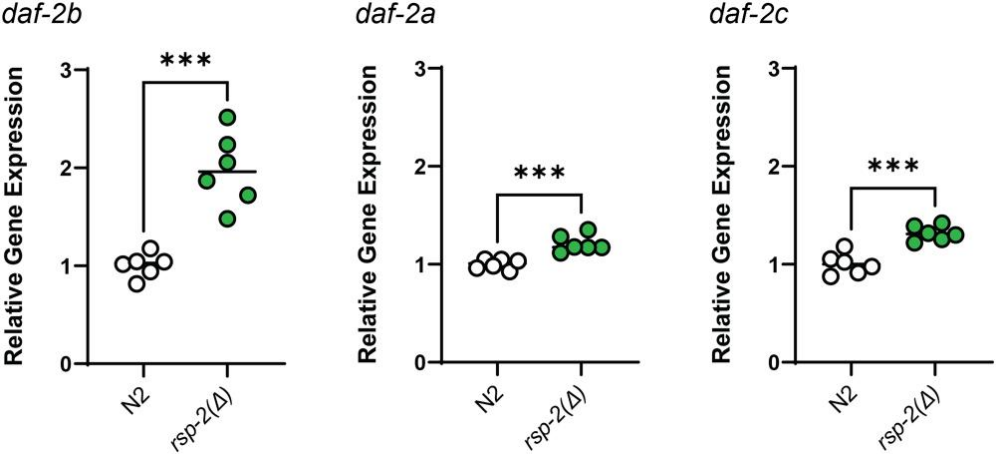
Genetic deletion of *rsp-2* increases expression of endogenous *daf-2b* transcripts. Relative gene expression of *daf-2b, daf-2a* and *daf-2c* transcripts in wild type and *rsp-2(Δ)* L1 animals. Data are derived from 6 independent populations and normalized to 2 reference genes. Student's *t*-test *** *P* < 0.0001).

The DAF-2B::tdTomato reporter indicates a capacity to generate the DAF-2B isoform but it does not demonstrate that the endogenous *daf-2b* transcript is increased by loss of *rsp-2.* We therefore used qPCR to verify that genetic loss of *rsp-2* also led to an increase in endogenous *daf-2b* expression. Consistent with the reporter data, we found that endogenous *daf-2b* transcripts were significantly elevated in *rsp-2(Δ)* L1 animals (Fig. 2). There was also an increase in *daf-2a* and *daf-2c* transcripts, but the magnitude of the increase was much less than that of *daf-2b* (Fig. 2).

### Dauer formation is associated with reduced RSP-2 expression

**Fig. 3.**
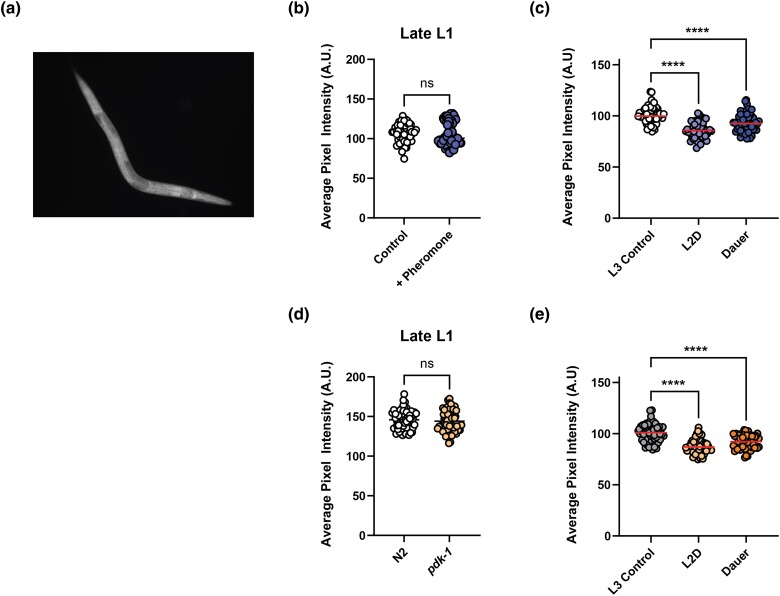
Expression of *rsp-2* is influenced by dauer formation. a) An *rsp-2* transcriptional reporter is expressed in all cells. b) Expression of an *rsp-2* transcriptional reporter is not different between ethanol-vehicle and pheromone-treated animals at the dauer decision stage in late L1. Pooled data from 3 independent replicates, Student's *t*-test *P* = n.s.. c) Expression of an *rsp-2* transcriptional reporter is reduced in pheromone-induced L2d and dauer animals compared with late L3 animals exposed to ethanol vehicle. Pooled data from 3 independent replicates, Tukey's multiple comparison test ****P < 0.0001. d) Expression of an *rsp-2* transcriptional reporter is not different between wild type N2 and *pdk-1* mutant animals at the dauer decision stage in late L1 at 26.5°C. Pooled data from 3 independent replicates Student's *t*-test *P* = n.s.. e) Expression of an *rsp-2* transcriptional reporter is reduced in *pdk-1* L2d and dauer animals compared with late L3 *pdk-1* animals. Pooled data from 3 independent replicates, Tukey's multiple comparison test ****P < 0.0001.

Gene expression studies have previously annotated *rsp-2* as a strongly regulated dauer gene, with expression reduced in dauers in a transforming growth factor-β (TGF-β) mutant background (Liu *et al*. 2004). To confirm the effect of dauer formation on *rsp-2* expression, we used CRISPR to generate a single copy transcriptional reporter in which an *rsp-2p::mNeonGreen::rsp-2 3′UTR* construct was inserted into a safe harbor site in chromosome I. In wild-type animals, fluorescence was broadly expressed in all tissues (Fig. 3a), consistent with previous *rsp-2* reporter strains (Kawano *et al*. 2000). There was no difference in fluorescence between vehicle and pheromone treated animals at the late L1 stage when the decision to enter dauer is being made (Fig. 3b). However, fluorescence was significantly lower in pheromone treated L2d and dauer larvae compared with L3 animals (Fig. 3c). Similarly, in a *pdk-1* mutant background, *rsp-2* fluorescence was not different at the late L1 dauer decision stage (Fig. 3d) but in L2d and dauer animals, there was a significant reduction compared with L3 animals (Fig. 3e), confirming that commitment into dauer is associated with reduced *rsp-2* expression.

### Loss of *rsp-2* influences pheromone-induced dauer entry

**Fig. 4.**
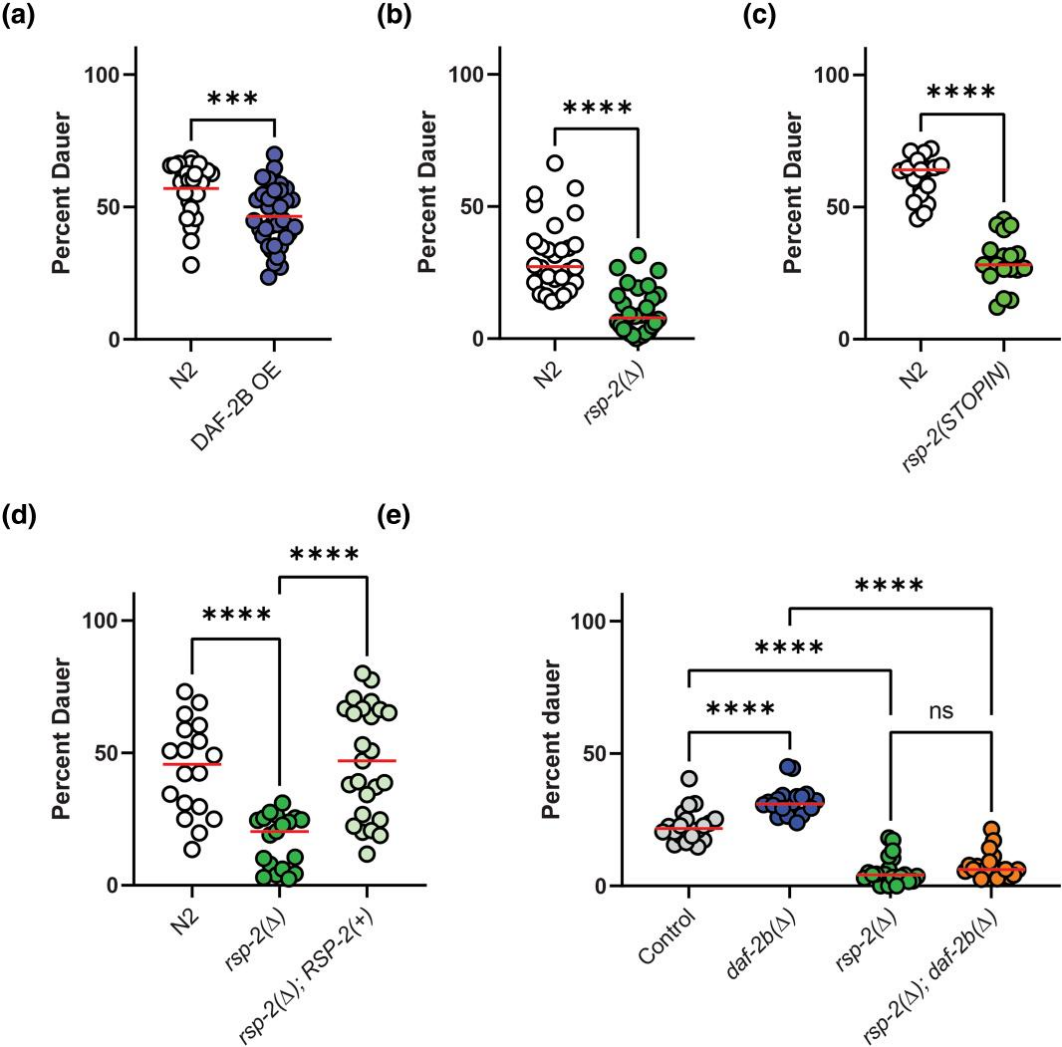
Genetic deletion of *rsp-2* suppresses pheromone-induced dauer formation. a) Overexpression of DAF-2B from the native DAF-2 promoter reduces pheromone-induced dauer formation in wild type animals. Pooled data from 3 independent replicates are redrawn from Martinez *et al* (2020), which is published under a CC BY 4.0 License. Student's *t*-test *** *P* < 0.001). b) *rsp-2(Δ)* reduces pheromone-induced dauer formation in wild type animals. Pooled data from 3 independent replicates, Student's *t*-test ****P < 0.0001. c) *rsp-2* STOPIN null mutation reduces pheromone-induced dauer formation. Pooled data from 2 independent replicates, Student's *t*-test ****P < 0.0001. d) Transgenic expression of RSP-2 rescues pheromone-induced dauer formation in *rsp-2(Δ)* mutants. Pooled data from 2 independent replicates and 3 independent transgenic lines, Tukey's multiple comparison test ****P < 0.0001. e) *rsp-2(Δ)* deletion suppresses dauer formation in *daf-2b(Δ)* mutants. Pooled data from 2 independent replicates, Tukey's multiple comparison test ****P < 0.0001.

Since loss of *rsp-2* leads to an increase in endogenous *daf-2b* transcripts, we hypothesized that *rsp-2(Δ)* mutants would exhibit phenotypes like those observed with DAF-2B overexpression. We previously showed that overexpression of DAF-2B from the native *daf-2* promoter decreased pheromone-induced dauer formation (Fig. 4a) (Martinez *et al*. 2020). In line with this, *rsp-2(Δ)* mutants exhibited reduced dauer entry in response to dauer pheromone (Fig. 4b), replicating the effect of DAF-2B overexpression. The deletion in *rsp-2(Δ)* mutants removes 984 bp of sequence starting in the latter half of exon 2 and extending into the 3′ untranslated region. The resulting protein product, if translated, would correspond to 91 amino acids of N-terminal sequence followed by a premature stop codon. Since the N-terminus of SR proteins contains the RRM, it is possible that the *rsp-2(Δ)* deletion encodes a truncated protein that retains some residual RNA binding capability activity and is therefore not a null mutant. To address this, we used CRISPR to introduce a 43 bp STOPIN cassette that has stop codons in every reading frame (Wang *et al*. 2018) into a PAM sequence 68 bp from the ATG start site using homology-directed repair (Paix *et al*. 2015; Wang *et al*. 2018). This results in a premature stop codon 33 amino acids from the translation start site, disrupting the RRM and therefore likely generates a true null mutant. After outcrossing, the *rsp-2(STOPIN)* mutant exhibited a similar reduction in pheromone-induced dauer formation as the *rsp-2(Δ)* mutant (Fig. 4c), suggesting that both alleles act as nulls. The reduced dauer entry phenotype in the *rsp-2(Δ)* background was reversed in transgenic animals expressing wild-type RSP-2 (Fig. 4d), confirming that the dauer phenotype is consequence of loss of *rsp-2* gene function. Although these data are consistent with elevated endogenous DAF-2B in the *rsp-2* mutants, the suppression of dauer entry by *rsp-2(Δ)* was maintained in *rsp-2(Δ); daf-2b(Δ)* double mutants (Fig. 4e), indicating that *rsp-2* functions downstream of, or in parallel to, *daf-2b* with respect to pheromone-induced dauer entry. Moreover, this result suggests that the suppression of pheromone-induced dauer formation by *rsp-2* deletion is not a consequence of increased *daf-2b* expression.

### Loss of *rsp-2* influences dauer formation in response to reduced insulin signaling

**Fig. 5.**
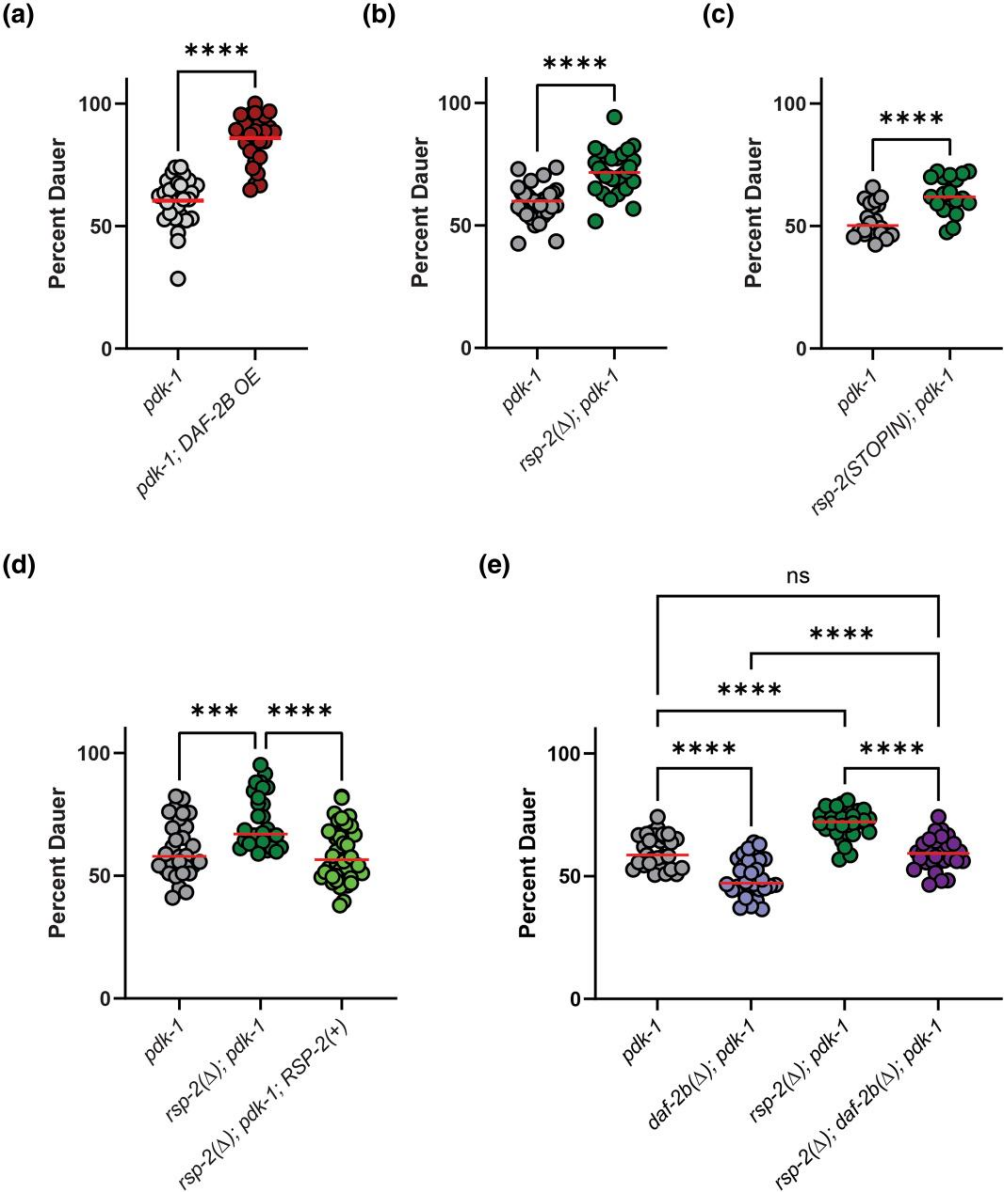
Genetic deletion of *rsp-2* enhances dauer formation in *pdk-1* mutants. a) Overexpression of DAF-2B from the native *daf-2* promoter increases dauer formation in *pdk-1* mutants. Pooled data from 3 independent replicates, Student's *t*-test ****P < 0.0001. b) *rsp-2(Δ)* deletion increases dauer formation in *pdk-1* mutants. Pooled data from 3 independent replicates, Student's *t*-test ****P < 0.0001. c) *rsp-2* STOPIN null mutation increases dauer formation in *pdk-1* mutants. Pooled data from 2 independent replicates, Student's *t*-test ****P < 0.0001. d) Transgenic expression of RSP-2 rescues dauer formation in *rsp*-2*(Δ)*; *pdk-1* deletion mutants. Pooled data from 3 independent replicates and 3 independent transgenic lines, Tukey's multiple comparison test ***P < 0.001, ****P < 0.0001. e) *daf-2b(Δ)* deletion partially suppresses the effect of *rsp-2(Δ)* on dauer formation in the *pdk-1* mutant background. Pooled data from 3 independent replicates, Tukey's multiple comparison test ****P < 0.0001.

In this study we used a hypomorphic mutant of *pdk-1* as our model for reduced insulin signaling. PDK-1 encodes the ortholog of human PDPK1 (3-phosphoinositide dependent protein kinase 1) and acts in the DAF-2 signal transduction pathway, downstream of DAF-2/IR and AGE-1/PI3K but upstream of AKT-1/Akt (Murphy and Hu 2013). In contrast to pheromone-induced dauer formation, overexpression of DAF*-*2B promotes dauer formation in models of reduced insulin signaling (Martinez *et al*. 2020), which was confirmed the *pdk-1* mutant background (Fig. 5a). Based on this, we hypothesized that *rsp-2* loss-of-function would enhance dauer formation in the insulin-signaling mutant *pdk-1(sa709)*. At a semi-permissive temperature, we observed increased dauer formation in both *rsp-2(Δ); pdk-1* and *rsp-2(STOPIN); pdk-1* double mutants (Fig. 5b and c). The increased dauer entry phenotype in the *rsp-2(Δ); pdk-1* double mutant background was reversed in transgenic animals expressing wild type RSP-2 (Fig. 5d), confirming that the dauer phenotype is a consequence of loss of *rsp-2* gene function.

To examine the requirement for *daf-2b* in this model of dauer entry, we generated an *rsp-2(Δ); daf-2b(Δ); pdk-1* mutant, along with the corresponding control strains. Loss of *daf-2b* in the *pdk-1* control background suppressed dauer formation (Fig. 5e) consistent with our previous observations (Martinez *et al*. 2020). In addition, the *rsp-2(Δ); pdk-1* control strain showed increased dauer formation, reinforcing the effect of *rsp-2(Δ)* in another genetic background. When both *rsp-2* and *daf-2b* were deleted together in the *pdk-1* control background dauer formation was suppressed relative to *rsp-2(Δ); pdk-1* but was still higher than *daf-2b(Δ); pdk-1* (Fig. 5e). These data suggest that in this model of dauer formation, *daf-2b* is epistatic to *rsp-2*, although *rsp-2* is only partially dependent on *daf-2b*.

### Loss of *rsp-2* influences dauer recovery

**Fig. 6.**
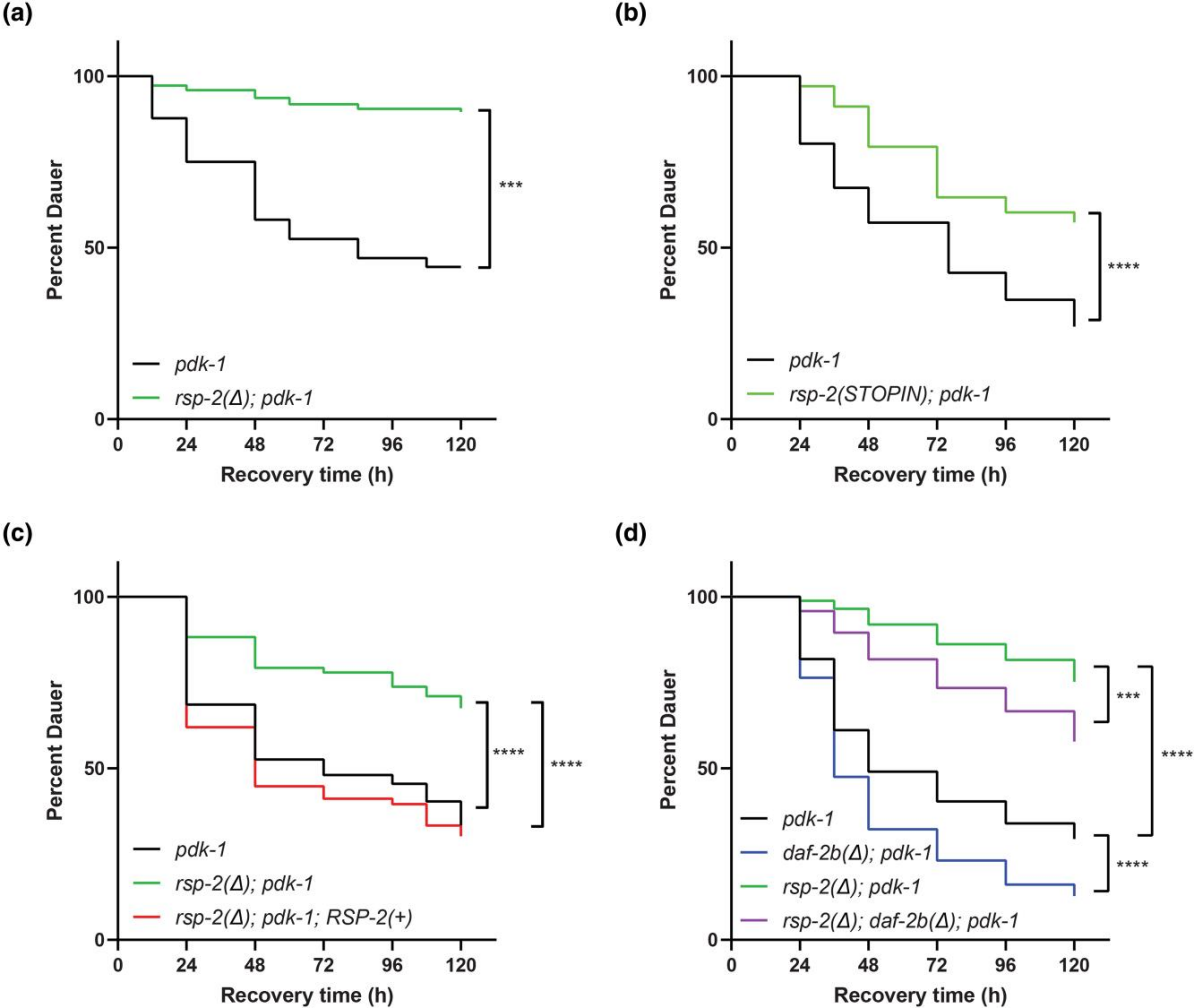
Genetic deletion of *rsp-2* inhibits dauer recovery. a) Recovery from dauer in *pdk-1* mutants is inhibited by *rsp-2(Δ)* deletion. Representative data from 3 independent trials, Log-rank test ****P < 0.0001. b) Recovery from dauer in *pdk-1* mutants is inhibited by *rsp-2* STOPIN null mutation. Representative data from 3 independent trials, Log-rank test ****P < 0.0001. c) Reduced dauer recovery in *rsp-2(Δ); pdk-1* mutants is rescued by transgenic expression of wild-type RSP-2. Representative data from 2 independent trials and 3 independent transgenic lines, Log-rank test *2(Δ); pdk-1* ****P < 0.0001. d) Recovery from dauer in *rsp-2(Δ); pdk-1* mutants is partially suppressed by *daf-2b(Δ).* Representative data from 3 independent trials, Log-rank test ***P < 0.001, ****P < 0.0001.

Overexpression of DAF-2B also slows recovery from the dauer stage in insulin-signaling mutants (Martinez *et al*. 2020). Consistent with this, we found that the *rsp-2(Δ)* and *rsp-2(STOPIN)* mutants in the *pdk-1* background conferred reduced recovery from the dauer stage (Fig. 6a and b), which was rescued in the *rsp-2(Δ)* mutant by transgenic expression of RSP-2 (Fig. 6c). The reduced dauer recovery phenotype of *rsp-2(Δ); pdk-1* double mutants was partially suppressed when *daf-2b* was deleted, suggesting that the effects of *rsp-2* deletion on dauer recovery in insulin signaling mutants is mediated in part by *daf-2b* (Fig. 6d).

### Loss of *rsp-2* increases lifespan in a *daf-2b* independent manner

**Fig. 7.**
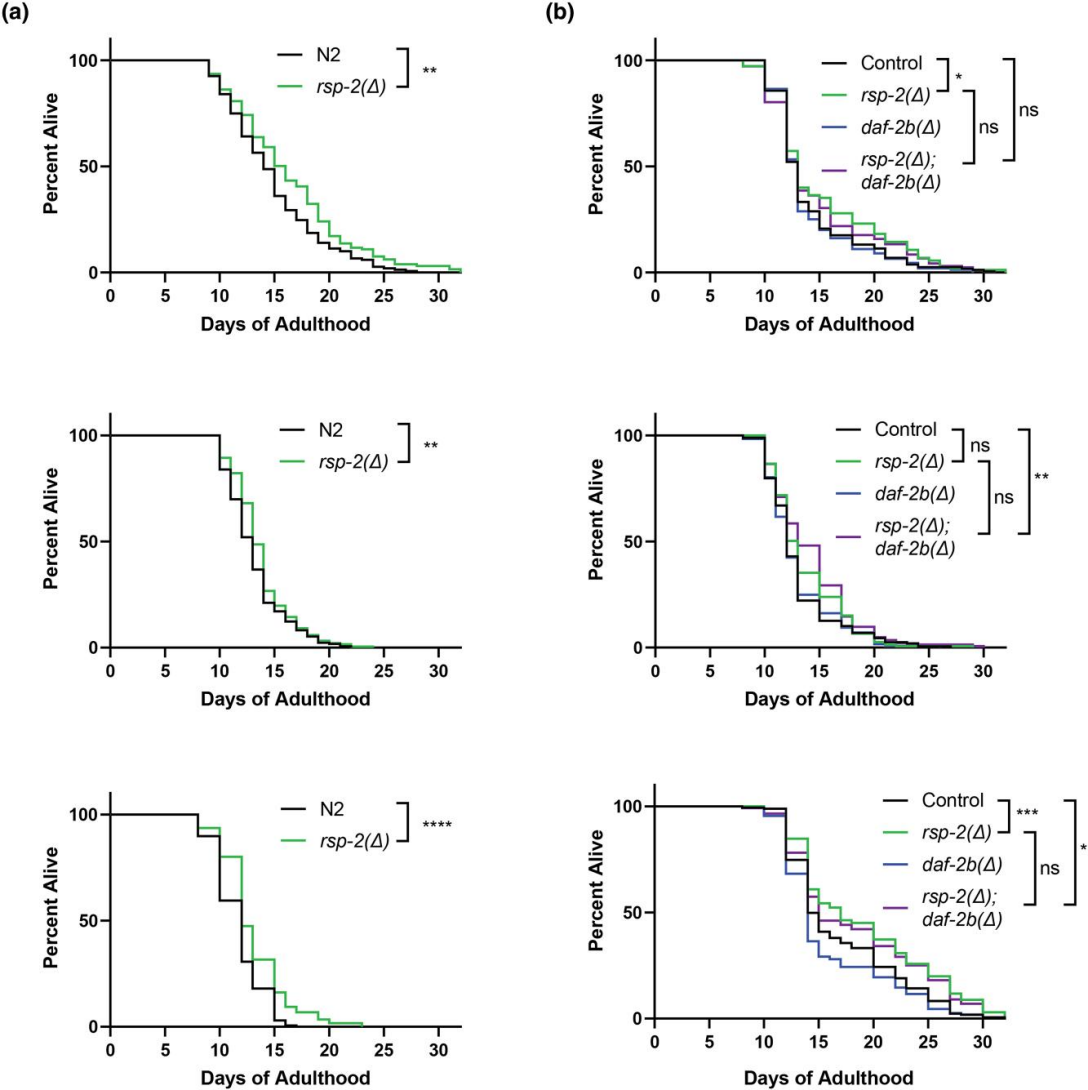
Genetic deletion of *rsp-2* extends lifespan independently of *daf-2b*. a) *rsp-2(Δ)* deletion increases lifespan. Data are replicate survival experiments carried out in the presence of 10μg/mL FUDR. Log-rank test ****P < 0.0001, **P < 0.01. b) Lifespan increase due to *rsp-2(Δ)* does not require *daf-2b.* Data are replicate survival experiments in the presence of 10μg/mL FUDR. Log-rank test ***P < 0.001, **P < 0.01, *P < 0.05.

Since overexpression of DAF-2B confers lifespan extension in adult animals (Martinez *et al*. 2020), we hypothesized that loss of *rsp-2* would also increase lifespan. The compound FUDR is used in *C. elegans* survival studies to inhibit progeny production. In the presence of FUDR, we found a reproducible increase in lifespan in *rsp-2* mutants compared with N2 animals (3/3 replicates, Fig. 7a; Supplementary Table 4 in Supplementary File 1). Since some studies have shown that FUDR may alter the lifespan response (Aitlhadj and Sturzenbaum 2010; Van Raamsdonk and Hekimi 2011), we also performed lifespan analysis in the absence of FUDR and observed a similar reproducible lifespan increase (3/3 replicates, Supplementary Fig. 2 and Supplementary Table 4 in Supplementary File 1). Based on this, subsequent lifespan analyses were performed in the presence of FUDR. To examine the requirement for *daf-2b* in mediating this lifespan increase, we examined *rsp-2(Δ); daf-2b(Δ)* double mutants. In this more complex genetic background (see methods), we observed increased lifespan in *rsp-2(Δ)* animals in 2/3 replicate experiments (Fig. 7b; Supplementary Table 4 in Supplementary File 1), reinforcing the idea that loss of *rsp-2* increases lifespan. Lifespan in *rsp-2(Δ); daf-2b(Δ)* mutants was not different from *rsp-2(Δ)* mutants in all 3 trials, and *rsp-2(Δ); daf-2b(Δ)* mutants were long-lived relative to control in 2/3 trials (Fig. 7b; Supplementary Table 4 in Supplementary File 1). These data indicate that the longevity of *rsp-2(Δ)* mutants is not mediated by *daf-2b*.

## Discussion

SR proteins are part of the core splicing machinery but are also involved in alternative splicing (Jeong 2017; Barberan-Soler *et al*. 2011) and polyadenylation site selection (Bradley *et al*. 2015). We previously identified a truncated isoform of the *C. elegans* IR, termed DAF-2B, that arises via alternative splicing (Martinez *et al*. 2020). In this study, we determined that RSP-2, a splicing factor from the SR protein family, is involved in regulating the expression of the *daf-2b* transcript. Loss of *rsp-2* not only increased expression of our fluorescent splicing reporter, but also increased expression of endogenous *daf-2b* transcripts, based on qPCR, indicating that the normal function of RSP-2 is to inhibit expression of DAF-2B.

The *daf-2b* transcript is predicted to be generated by activation of an alternative polyadenylation site in the intron after exon 11.5 that inhibits splicing from the exon 11.5 5′ splice site. In contrast, the full-length *daf-2c* transcript is generated when this alternative polyadenylation site is inhibited, leading to splicing from exon 11.5 to exon 12. Given the reciprocal mechanisms by which these 2 transcripts arise, we considered the possibility that there could be an inverse relationship between the expression of *daf-2b* and *daf-2c* in response to deletion of *rsp-2.* Specifically, if the function of RSP-2 is to inhibit expression of *daf-2b,* then it is possible that it also functions to promote expression of *daf-2c*. If this were the case, loss of *rsp-2* would not only be associated with an increase in *daf-2b* transcripts but also a reduction in *daf-2c* transcripts. However, this is not supported by our qPCR data. Loss of *rsp-2* increased *daf-2b* transcripts but did not decrease *daf-2c* transcripts, suggesting that *daf-2b,* but not *daf-2c,* is a target of RSP-2. It is not clear why there is a small increase in *daf-2a/c* transcripts, but one possibility is that the animal attempts to increase insulin sensitivity by increasing expression of the full-length receptors to balance the insulin resistance conferred by increased DAF-2B.

The increase in *daf-2b* transcripts following knockdown of *rsp-2* suggests that *rsp-2* mutants represent a model of physiological overexpression of DAF-2B. Like DAF-2B overexpression, we found that *rsp-2* mutants modified dauer phenotypes in 2 different contexts, as well as influencing dauer recovery and lifespan. Despite these similarities, only dauer entry and dauer recovery under conditions of reduced insulin signaling were dependent on DAF-2B while pheromone-induced dauer formation and lifespan did not require DAF-2B. With respect to lifespan, we previously found that DAF-2B overexpression confers large increases in lifespan, depending on the promotor that was used for transgene expression. Although *rsp-2* mutants also conferred lifespan extension, the effect was much smaller and was independent of DAF-2B, suggesting that RSP-2 functions to maintain normal lifespan through targets other than DAF-2B.

In our previous study, we observed that overexpression of DAF-2B suppressed pheromone-induced dauer formation, but enhanced dauer formation in hypomorphic insulin signaling mutants (Martinez *et al*. 2020). Mechanistically, this apparent paradox can be explained in the context of differences in the prevailing insulin milieu. Pheromone treatment has been shown to downregulate the expression of agonist insulin peptides, such as DAF-28 and INS-6 (Li *et al*. 2003; Cornils *et al*. 2011), while the expression of the antagonist ILP INS-1 (Pierce *et al*. 2001) remains stable in response to pheromone (Cornils *et al*. 2011). The net effect of this would be a relative increase in antagonistic insulin peptides. Expression of another antagonist insulin peptide, INS-18, is increased in dauers (Matsunaga *et al*. 2012), and overexpression increases pheromone induced dauer formation (Matsunaga *et al*. 2012; Martinez *et al*. 2020). Conversely, overexpression of DAF-2B reduces pheromone-induced dauer formation and suppresses the effect of INS-18 overexpression (Martinez *et al*. 2020), suggesting that DAF-2B can sequester antagonist ILPS to promote insulin signaling and reproductive growth. On the other hand, reduced insulin signaling in hypomorphic insulin signaling mutants leads to an elevation of agonist insulin peptides (Fernandes de Abreu *et al*. 2014), presumably as part of a negative feedback loop that tries to maintain insulin sensitivity in the face of insulin resistance at the receptor level. Increased expression of DAF-2B sequesters insulin agonists, thereby reducing DAF-2 activation and promoting dauer formation (Martinez *et al*. 2020).

**Fig. 8.**
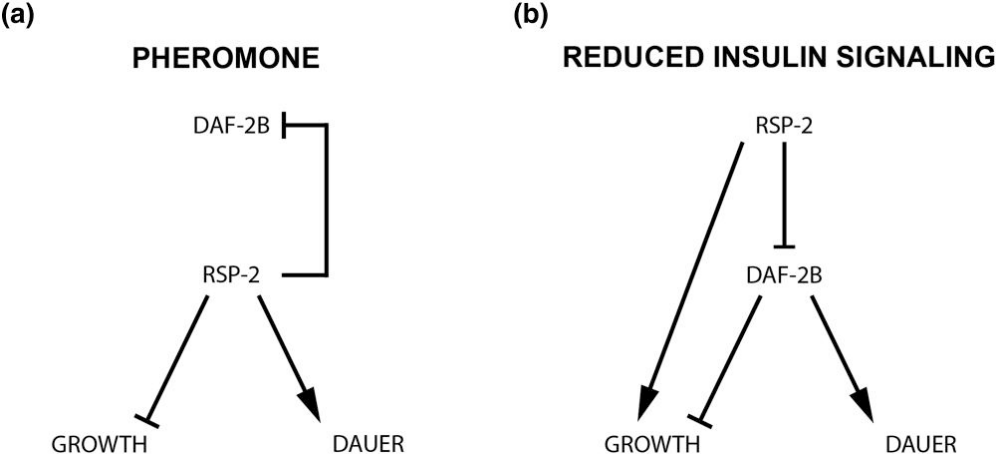
Model describing the role of RSP-2 in dauer formation. a) In the pheromone model of dauer formation, *rsp-2* is epistatic to *daf-2b* indicating that the wild-type function of RSP-2 is to inhibit reproductive growth and promote dauer formation independently of DAF-2B. b) In the reduced insulin signaling model, *daf-2b* is epistatic to *rsp-2,* but the effect is incomplete. This indicates that the wild type function of RSP-2 in this context is to promote reproductive growth in part by inhibiting the expression of DAF-2B.

In this study, we found that deletion of *rsp-2* maintained these context-specific effects, as well as reducing dauer recovery in *pdk-1* mutants. In the presence of pheromone, *rsp-2* deletion promoted reproductive growth suggesting that the wild type activity of RSP-2 is to promote dauer formation and inhibit reproductive growth (Fig. 8a). Even though loss of *rsp-2* increases DAF-2B, the epistasis data in Fig. 4e indicates that the effect of RSP-2 on pheromone induced dauer formation is epistatic to DAF-2B (Fig. 8a). This suggests that RSP-2 influences pheromone-induced dauer formation independently of DAF-2B. Conversely, in the reduced insulin signaling background of *pdk-1* mutants, loss of *rsp-2* enhanced dauer formation in a manner that was at least partially dependent on DAF-2B. This suggests that under these conditions, the wild type function of RSP-2 is to promote reproductive growth which it achieves in part by inhibiting DAF-2B (Fig. 8b).

These observations suggest that RSP-2 can take on a pro-dauer activity in the pheromone model and a pro-growth activity in the reduced insulin signaling model. This apparent paradox could be explained by differences in the levels of antagonist and agonist ILPs as well as other signaling inputs. For example, the conditions employed for pheromone-induced dauer formation (reduced food, elevated temperature as well as pheromone extract) target multiple signaling pathways in addition to insulin signaling, such as TGF-B and target of rapamycin (TOR) signaling. Collectively, these inputs may lead to changes in posttranslational modifications of RSP-2 that influence its activity. In this respect, it is well established that the activity of SR proteins is regulated by phosphorylation (Patel *et al*. 2005; Aubol *et al*. 2013; Zhou and Fu 2013) and of particular relevance is the observation that the mammalian ortholog of RSP-2, Srp40/SRSF5, is insulin responsive and can mediate changes in alternative splicing in an Akt-dependent manner (Patel *et al*. 2005). It is notable that an unbiased assessment of the *C. elegans* phosphoproteome identified 2 phosphopeptides from RSP-2 that contain predicted phosphorylation motifs for kinases including GSK3 and AKT, among others (Zielinska *et al*. 2009). It is therefore an intriguing possibility that the phosphorylation status of RSP-2 is coordinated by different kinases which in turn may influence the activity of RSP-2 with respect to dauer formation. Future studies aimed at manipulating these potential phosphorylation sites will be required to determine the role that phosphorylation of RSP-2 plays in modifying life history traits such as dauer formation.

Expression of *rsp-2* has been reported to be down-regulated during the process of dauer formation (Liu *et al*. 2004). Using a transcriptional reporter, we confirmed that *rsp-2* expression is decreased in animals that are committed to dauer formation. However, at the dauer decision stage in late L1 larvae, there was no difference in *rsp-2* expression between controls and animals destined to become dauers. One explanation for this is that RSP-2 may not be instructive in the dauer formation process but instead may be involved in changing patterns of splicing after commitment to the dauer program. RSP-2 may still be involved in the dauer decision but its activity is not regulated at the transcriptional level. In this latter respect, changes in phosphorylation status may initiate differential splicing patterns. Alternatively, reduced expression of RSP-2 during dauer formation may be a consequence of systemic changes induced the dauer program.

In conclusion, we have determined that RSP-2, a member of the *C. elegans* SR protein family, influences expression of the truncated IR DAF-2B. Although loss of function mutations in *rsp-2* generates phenotypes similar to DAF-2B overexpression, we find that RSP-2 functions independently of DAF-2B for some phenotypes and is partially dependent on DAF-2B for others.

## Supplementary Material

jkad064_Supplementary_Data

## Data Availability

Worm strains and reagents are available upon request. Supplementary materials include Supplementary Figs. 1 and 2 and Supplementary Tables 1–4 in Supplementary File 1. All raw data, including replicates not shown in figures, are available in Supplementary File 2. Supplemental material available at G3 online.
