# Supplementary material for: The SR protein RSP-2 influences expression of the truncated insulin receptor DAF-2B in Caenorhabditis elegans
Source: G3 (Bethesda). 2023 Mar 26;13(6):jkad064. doi: 10.1093/g3journal/jkad064 (PMC10234397; doi:10.1093/g3journal/jkad064)
Supplement: jkad064_Supplementary_Data [file jkad064_supplementary_data.zip › File_S1_G3-2022-403970.docx]

## Figure S1. RNAi of *rsp-2* increases expression of a *daf-2b* splicing reporter

Quantitation of DAF-2B::tdTomato expression through development in worms treated with control and *rsp-2* RNAi. Data are derived from 20 images per genotype at each developmental stage. Student’s t-test ****p<0.0001.


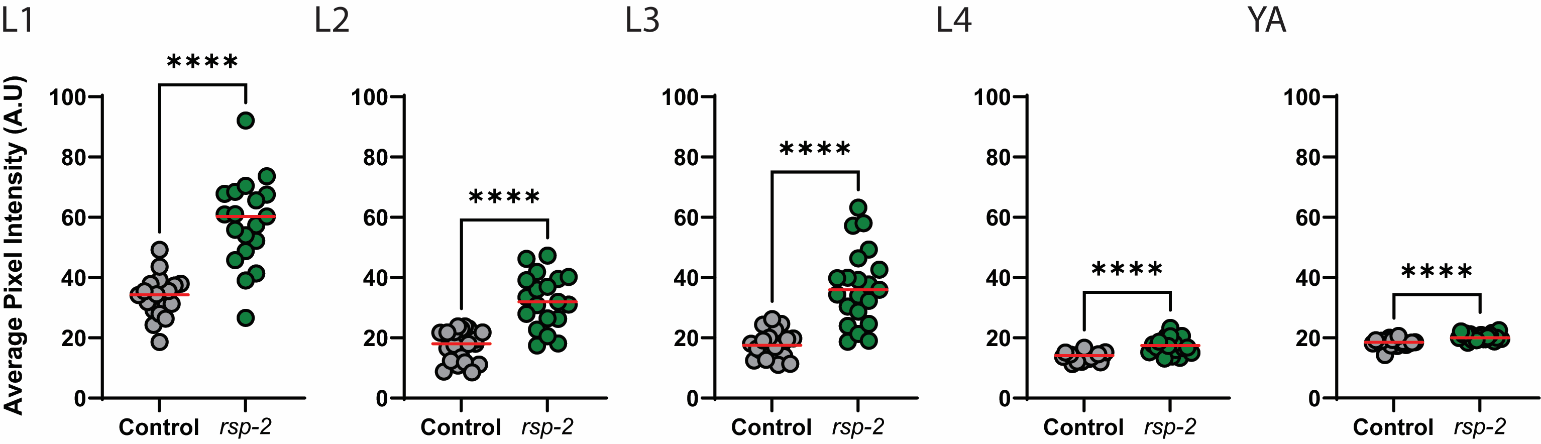


## Figure S2. Genetic deletion of *rsp-2* extends lifespan in the absence of FUDR

**(A)** *rsp-2(Δ)* deletion increases lifespan. Data are replicate survival experiments carried out in the absence of FUDR. Log-rank test ****p<0.0001, **p<0.01.


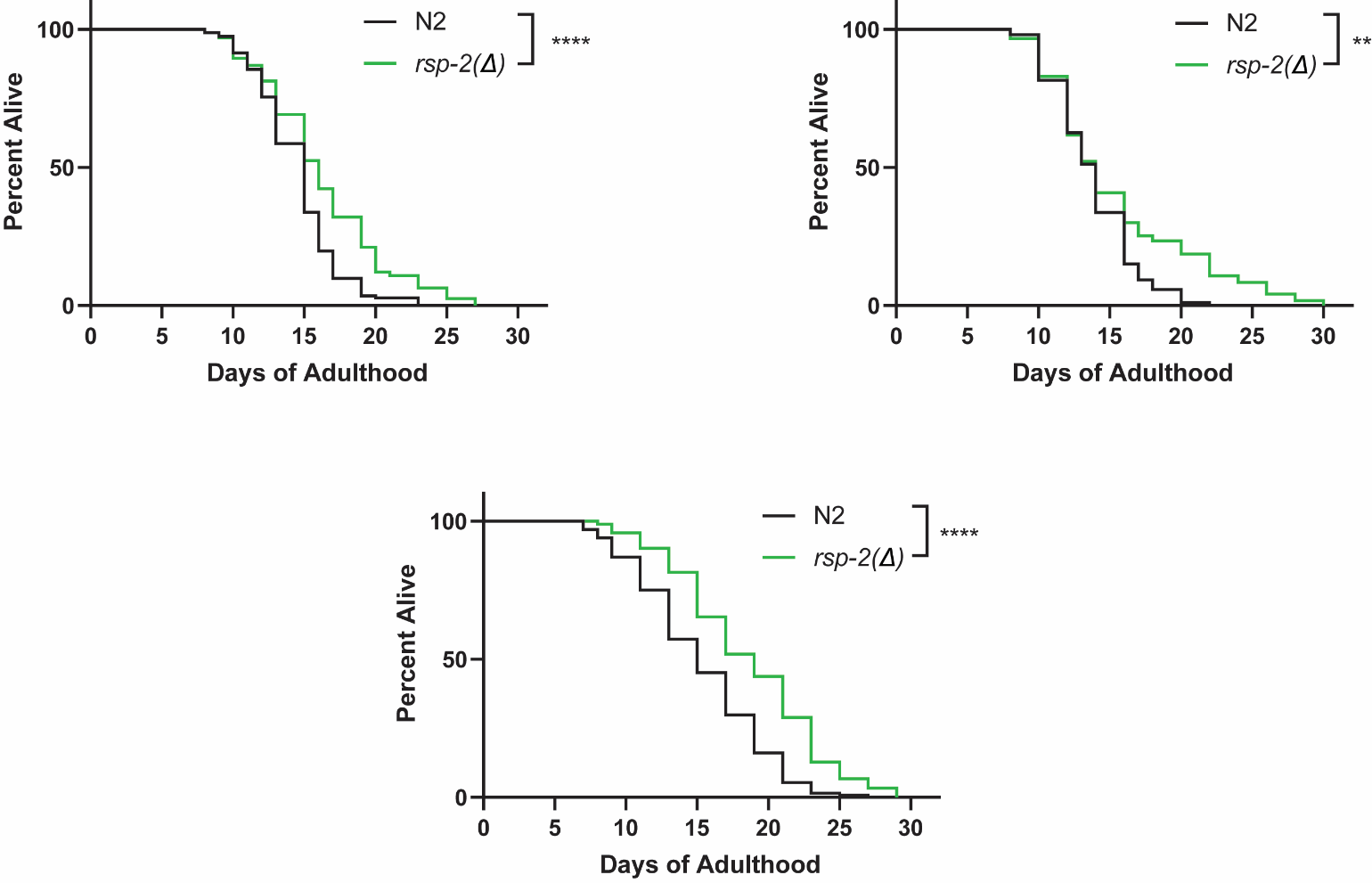


## Table S1. List of primers and oligos used in this study.

| **Purpose** | **Primers** |
| --- | --- |
| *rsp-2* genomic rescue |  |
| - *rsp-2* promoter | 1 ACATGCATGCGCGAATGAATGAATGGCCTGTATCACAG |
|  | 2 ATTACCGGTGTTTAGAGGTCTGAAATAAGTGAAGAATGAG |
| - *rsp-2* genomic | 3 ATTACCGGTATGGTTCGTGTCTACATCGGTC |
|  | 4 CGGGGTACCTTATGGAGATGCAGATCCGGAACG |
| Plasmid sequencing | 5 AGCACAGGGAGAAAGAGCATGTAG |
| *rsp-2(ok639)* genotyping | 6 CGCGGATATGGAAAGCTGTC |
|  | 7 GTCGTCATGGGAAGTGAAGC |
|  | 8 TGTCCGTTCTTCCCGACTAC |
| *daf-2bc(Δ)* genotyping | 9 TGCATTTGAGAATAAGCTGTTGG |
|  | 10 TATGCCTGCTCCAAGCCTAT |
|  | 11 CGCATAAAGTGTGTAATGCTTCAAAT |
| MosSCI genotyping | 12 CGCTACTTACCGGAAACCAA |
|  | 13 TTTCTCAGTTGTGATACGGTTTTT |
|  | 14 CAATTCATCCCGGTTTCTGT |
|  | 15 TCTGGCTCTGCTTCTTCGTT |
| *dpy-10* crRNA | 16 GCUACCAUAGGCACCACGAGGUUUUAGAGCUAU GCUGUUUUG (Paix et al. 2015) |
| *dpy-10* HDR template | 17 CACTTGAACTTCAATACGGCAAGATGAGAATGACTG GAAACCGTACCGCATGCGGTGCCTATGGTAGCGGAGCTTCACATGGCTTCAGACCAACAGCCTAT (Paix et al. 2015) |
| *rsp-2* CRISPR STOPIN crRNA | 18 GAUGUCGAGCACUUCUUCCG |
| *rsp-2* CRISPR STOPIN HDR template | 19 CAAATAGAGCATCTGATCGTGATGTCGAGCACTTCTTGGGAAGTT TGTCCAGAGCAGAGGTGACTAAGTGATAAGCTAGCCCGCGGATATGGAAAGCTGTCTGATGTCATAATG |
| *rsp-2* CRISPR STOPIN genotyping | 20 GCGAATGAATGAATGGCCTGTATCACAG |
|  | 21 GCTTATCACTTAGTCACCTCTGCTC (Wang et al. 2018) |
|  | 22 ATGGTTCGTGTCTACATCGGTC |
|  | 23 CTGCCAACTGTAACGAGTAGACAAA |
| ttTi4348 cRNA | 24 UUUUGUCAAAAGAAGAGACA (Silva-Garcia et al. 2019) |
| *prsp-2* with overhangs | 25 TCTCACTCTGATGAGCGTATCTATCAAGTCCTTGTGCGAATGAATG AATGGC CTGTATCACAG |
|  | 26 GAAGCCATGTTATCCTCTTCTCCCTTCGACACCATGTTTAGAGGTC TGAAATAAGTGAAGAATGAG |
| *mNeonGreen* with overhangs | 27 TTTTCTCATTCTTCACTTATTTCAGACCTCTAAACATGGTGTCGAAG GGAGA AGA |
|  | 28 GAAATTTCACATGAGAGGTGCAATGATAAATAAAACTACTTGTAGA GTTCATCCATTCCCAT |
| *rsp-2* 3’UTR with overhangs | 29 CAGATGTGATGGGAATGGATGAACTCTACAAGTAGTTTTATTTATC ATTGCACCTCTC |
|  | 30 TCGGCGATTTCTTTGAAGTTTTGTCAAAAGAAGAGTTTGCATCCAA ATTGTTTTATTG |
| *rsp-2 reporter genotyping* | 31 TAAGCTTCTCACACCTTTTCTCTCG |
|  | 32 CCTCCCCTCATCTCAATTATCCC |
|  | 33 ATGGGAATGGATGAACTCTACAAGTAG |
| *rsp-2 reporter sequencing* | 34 CTCGTATGTGTATCGGTAGTTCACTGT |
| qPCR |  |
| *act-1* | 35 GCTGGACGTGATCTTACTGATTACC *(Hoogewijs et al. 2008)* |
|  | 36 GTAGCAGAGCTTCTCCTTGATGTC |
| *ama-1* | 37 CCTACGATGTATCGAGGCAAA *(Hoogewijs et al. 2008)* |
|  | 38 CCTCCCTCCGGTGTAATAATG |
| *cdc-42* | 39 CTGCTGGACAGGAAGATTACG *(Hoogewijs et al. 2008)* |
|  | 40 CTCGGACATTCTCGAATGAAG |
| *pmp-3* | 41 GTTCCCGTGTTCATCACTCAT *(Hoogewijs et al. 2008)* |
|  | 42 ACACCGTCGAGAAGCTGTAGA |
| *iscu-1* | 43 GTCGCTTCAAATCAGTTCAGC *(Hoogewijs et al. 2008)* |
|  | 44 GTTCTTGTCAAGTGATCCGACA |
| *snb-1* | 45 CCGGATAAGACCATCTTGACG *(Martinez et al. 2017)* |
|  | 46 GACGACTTCATCAACCTGAGC |
| *rps-2* | 47 CTCGAGGAGATCTACCTCAACTCC |
|  | 48 TACGTTGTCCGGCAGTGG |
| *rps-23* | 49 TCGTTCTTGAAAAGATCGGTG |
|  | 50 GAATATCTCCGACGGCGTG |
| *daf-2a* | 51 GACTATGATGGGTCACGAAGATC |
|  | 52 ACTCGCATCGTGTCTCTCG |
| *daf-2b* | 53 GGATGAGGTTTTAATGCCGAGATG |
|  | 54 GCATTACAGACATCATCAAATCATGC |
| *daf-2c* | 55 GCAGCCTTGGTGGAATTCAT |
|  | 56 GAAATATGGATCAGCGGCTTC |

## Table S2. List of plasmids used in this study

| **Name** | **Description** | **Comments** |
| --- | --- | --- |
| pMGL99 | pPD49.26 + GFP | GFP expression with MCS |
| pMGL115 | pPD49.26 *myo-3p::GFP::unc-54 UTR* | Muscle GFP expression |
| pMGL224 | pPD49.26 *rsp-2p::RSP-2::unc-54 UTR* | Native RSP-2 expression |
| pDG553 | pENTRslot2 mNeonGreen_stop | (Hostettler et al. 2017) |

## Table S3. List of strains used in this study

| **Strain** | **Genotype** | **Source** | **Description** |
| --- | --- | --- | --- |
| N2 | Wild type | CGC | N2 Bristol |
| JT709 | *pdk-1(sa709)* | CGC |  |
| VC463 | *rsp-2(ok639)* | CGC |  |
| MGL371 | *jluIs15[daf-2p::DAF-2bexon-11.5::tdTomato + rol-6(+)]* | *(Martinez et al. 2020)* | Integrated *daf-2b* splicing reporter |
| MGL297 | *jluSi3[daf-2p::DAF-2C + unc-119(+)]* | *(Martinez et al. 2020)* | *daf-2b(Δ)* control strain |
| MGL302 | *jluSi3[daf-2p::DAF-2C + unc-119(+)]; daf-2(jlu1)* | *(Martinez et al. 2020)* | *daf-2b(Δ)* |
| MGL372 | *rsp-2(ok639)* | This study | 5x outcross |
| MGL460 | Wild type | This study | Derived from 5x outcross of MGL372 |
| MGL373 | *rsp-2(ok639); jluIs15[daf-2p::DAF-2bexon-11.5::tdTomato + rol-6(+)]* | This study | *rsp-2* deletion in *daf-2b* splicing reporter |
| MGL374 | *pdk-1(sa709)* | This study | 3x outcross |
| MGL375 | *rsp-2(ok639); pdk-1(sa709)* | This study |  |
| MGL461 | *pdk-1(sa709)* | This study | Derived from MGL372 and MGL274 cross |
| MGL376 | *rsp-2(ok639) jluSi3[daf-2p::DAF-2C + unc-119(+)]* | This study | *rsp-2* deletion + *daf-2b(Δ)* control background |
| MGL377 | *rsp-2(ok639) jluSi3[daf-2p::DAF-2C + unc-119(+)]; daf-2(jlu1)* | This study | *rsp-2 deletion + daf-2b(Δ)* |
| MGL378 | *jluSi3[daf-2p::DAF-2C + unc-119(+)]; pdk-1(sa709)* | This study | *daf-2b(Δ)* control strain in *pdk-1* background |
| MGL379 | *rsp-2(ok639) jluSi3[daf-2p::DAF-2C + unc-119(+)]; daf-2(jlu1); pdk1(sa709)* | This study | *rsp-2(Δ); daf-2b(Δ); pdk-1* |
| MGL380 | *rsp-2(ok639) jluSi3[daf-2p::DAF-2C + unc-119(+)]; pdk-1(sa709)* | This study | *rsp-2; pdk-1* in *daf-2b(Δ)* control background |
| MGL381 | *jluSi3[daf-2p::DAF-2C + unc-119(+)]; daf-2(jlu1); pdk-1(sa709)* | This study | *daf-2b(Δ)* in *pdk-1* background |
| MGL382 | *rsp-2(ok639); jluEx185[rsp-2p::RSP-2 + myo-3p::GFP]* | This study | *rsp-2* deletion + RSP-2 genomic rescue |
| MGL383 | *rsp-2(ok639); jluEx186[rsp-2p::RSP-2 + myo-3p::GFP]* |  |  |
| MGL384 | *rsp-2(ok639); jluEx187[rsp-2p::RSP-2 + myo-3p::GFP]* |  |  |
| MGL385 | *rsp-2(ok639); pdk-1(sa709); jluEx188[rsp-2p::RSP-2 + myo-3p::GFP]* | This study | *rsp-2; pdk-1* + RSP-2 genomic rescue |
| MGL386 | *rsp-2(ok639); pdk-1(sa709); jluEx189[rsp-2p::RSP-2 + myo-3p::GFP]* |  |  |
| MGL387 | *rsp-2(ok639); pdk-1(sa709); jluEx190[rsp-2p::RSP-2 + myo-3p::GFP]* |  |  |
| MGL400 | *rsp-2(jlu14)* | This study | *rsp-2* STOPIN null, 2x outcross |
| MGL401 | *rsp-2(jlu14); pdk-1(sa709)* | This study | *rsp-2* STOPIN null in *pdk-1* background |
| MGL406 | *jluSi5*(*rsp-2p::mNeonGreen::rsp-2 3’UTR) I* | This study | *rsp-2p* transcriptional reporter |
| MGL407 | *jluSi5*(*rsp-2p::mNeonGreen::rsp-2 3’UTR) I; pdk-2(sa709)* | This study | *rsp-2p* transcriptional reporter in *pdk-1* background |
| MGL462 | *jluEx186(daf-2p::DAF-2B + myo-2p::tdTomato)* | This study | Extrachromosomal array expressing DAF-2B from native *daf-2* promoter |
| MGL450 | *jluIs18*(*daf-2p::DAF-2B + myo-2p::tdTomato)* | This study | Integrated DAF-2B overexpresser |
| MGL451 | *jluIs18(daf-2p::DAF-2B + myo-2p::tdTomato) pdk-1(sa709)* | This study | Integrated DAF-2B overexpresser in *pdk-1* mutant backgorund |

## Table S4. Summary data for all replicates of lifespan experiments.

| **Genotype** | **Trial** | **Median survival** | **Deaths**  **(censored)** | **P value** |
| --- | --- | --- | --- | --- |
| N2 | 1 | 15 | 146(20) |  |
| *rsp-2(ok639)* |  | 16 | 157(18) | P<0.0001 |
| N2 | 2 | 14 | 88(28) |  |
| *rsp-2(ok639)* |  | 14 | 169(20) | P<0.01 |
| N2 | 3 | 15 | 139(57) |  |
| *rsp-2(ok639)* |  | 19 | 150(40) | P<0.0001 |
| N2 + 10μg/ml FUDR | 1 | 14 | 159(51) |  |
| *rsp-2(ok639)* + 10μg/ml FUDR |  | 16 | 153(57) | P<0.01 |
| N2 + 10μg/ml FUDR | 2 | 13 | 185(25) |  |
| *rsp-2(ok639)* + 10μg/ml FUDR |  | 13 | 190(20) | P<0.01 |
| N2 + 10μg/ml FUDR | 3 | 12 | 168(12) |  |
| *rsp-2(ok639)* + 10μg/ml FUDR |  | 12 | 133(27) | P<0.0001 |
| N2 control + 10μg/ml FUDR | 1 | 13 | 163(17) |  |
| *rsp-2(Δ)* + 10μg/ml FUDR |  | 13 | 168(12) | P<0.05 vs N2 control |
| *daf-2b(Δ)* + 10μg/ml FUDR |  | 13 | 163(17) |  |
| *rsp-2(Δ); daf-2b(Δ)* + 10μg/ml FUDR |  | 13 | 169(11) | ns vs N2 control  ns vs *rsp-2(Δ)* |
| N2 control + 10μg/ml FUDR | 2 | 12 | 161(19) |  |
| *rsp-2(Δ)* + 10μg/ml FUDR |  | 13 | 162(18) | ns vs N2 control |
| *daf-2b(Δ)* + 10μg/ml FUDR |  | 12 | 152(28) |  |
| *rsp-2(Δ); daf-2b(Δ)* + 10μg/ml FUDR |  | 13 | 151(29) | P<0.01 vs N2 control  ns vs *rsp-2(Δ)* |
| N2 control + 10μg/ml FUDR | 3 | 14 | 171(9) |  |
| *rsp-2(Δ)* + 10μg/ml FUDR |  | 17 | 149(31) | P<0.001 vs N2 control |
| *daf-2b(Δ)* + 10μg/ml FUDR |  | 14 | 168(12) |  |
| *rsp-2(Δ); daf-2b(Δ)* + 10μg/ml FUDR |  | 15 | 105(15) | P<0.05 vs N2 control  ns vs *rsp-2(Δ)* |

Literature Cited

Hoogewijs, D., K. Houthoofd, F. Matthijssens, J. Vandesompele, and J.R. Vanfleteren, 2008 Selection and validation of a set of reliable reference genes for quantitative sod gene expression analysis in C. elegans. *BMC Mol Biol* 9:9.

Hostettler, L., L. Grundy, S. Kaser-Pebernard, C. Wicky, W.R. Schafer *et al.*, 2017 The Bright Fluorescent Protein mNeonGreen Facilitates Protein Expression Analysis In Vivo. *G3 (Bethesda)* 7 (2):607-615.

Martinez, B.A., D.A. Petersen, A.L. Gaeta, S.P. Stanley, G.A. Caldwell *et al.*, 2017 Dysregulation of the Mitochondrial Unfolded Protein Response Induces Non-Apoptotic Dopaminergic Neurodegeneration in C. elegans Models of Parkinson's Disease. *J Neurosci* 37 (46):11085-11100.

Martinez, B.A., P. Reis Rodrigues, R.M. Nunez Medina, P. Mondal, N.J. Harrison *et al.*, 2020 An alternatively spliced, non-signaling insulin receptor modulates insulin sensitivity via insulin peptide sequestration in C. elegans. *Elife* 9.

Paix, A., A. Folkmann, D. Rasoloson, and G. Seydoux, 2015 High Efficiency, Homology-Directed Genome Editing in Caenorhabditis elegans Using CRISPR-Cas9 Ribonucleoprotein Complexes. *Genetics* 201 (1):47-54.

Silva-Garcia, C.G., A. Lanjuin, C. Heintz, S. Dutta, N.M. Clark *et al.*, 2019 Single-Copy Knock-In Loci for Defined Gene Expression in Caenorhabditis elegans. *G3 (Bethesda)* 9 (7):2195-2198.

Wang, H., H. Park, J. Liu, and P.W. Sternberg, 2018 An Efficient Genome Editing Strategy To Generate Putative Null Mutants in Caenorhabditis elegans Using CRISPR/Cas9. *G3 (Bethesda)* 8 (11):3607-3616.
